# Specific Compositions of *Cannabis sativa* Compounds Have Cytotoxic Activity and Inhibit Motility and Colony Formation of Human Glioblastoma Cells In Vitro

**DOI:** 10.3390/cancers13071720

**Published:** 2021-04-05

**Authors:** Hadar Peeri, Nurit Shalev, Ajjampura C. Vinayaka, Rephael Nizar, Gila Kazimirsky, Dvora Namdar, Seegehalli M. Anil, Eduard Belausov, Chaya Brodie, Hinanit Koltai

**Affiliations:** 1 Institute of Plant Science, Agriculture Research Organization, Volcani Institute, Rishon LeZion 7505101, Israel; hadarpeeri10@gmail.com (H.P.); nuritsh@volcani.agri.gov.il (N.S.); ac.vinayaka@gmail.com (A.C.V.); dvoran@volcani.agri.gov.il (D.N.); aniganapath@gmail.com (S.M.A.); eddy@volcani.agri.gov.il (E.B.); 2 The Mina and Everard Goodman Faculty of Life Sciences, Bar-Ilan University, Ramat Gan 5290002, Israel; rephael92@gmail.com (R.N.); Gila.kazimirsky@biu.ac.il (G.K.); chaya@brodienet.com (C.B.); 3 Davidson Laboratory of Cell Signaling and Tumorigenesis, Hermelin Brain Tumor Center, Henry Ford Hospital, Detroit, MI 48202, USA

**Keywords:** glioblastoma, cannabis, phytocannabinoids, apoptosis, F-actin, cytotoxicity, cell cycle, cell migration, endoplasmic reticulum stress, neurospheres, glioma stem cells

## Abstract

**Simple Summary:**

Glioblastoma multiforme (GBM) is the most frequent, invasive, and lethal subtype of glioma brain tumors. Cannabis is commonly used for medical treatment, and individual phytocannabinoids have been shown to trigger GBM cell death. However, cannabis contains hundreds of different compounds, and the optimal combinations of molecules with anti-GBM activity are unknown. Here, we identified fractions from a cannabis strain that substantially reduced human GBM cell viability and motility. The fractions also reduced the ability of GBM cells to form colonies in 2 and 3-dimensional models, suggesting that the cannabis treatments may have the potential for preventing the formation of GBM neurospheres associated with the high resistance to current therapies. Importantly, these compounds also induced cell death in glioma stem cells derived from tumor specimens. The effectiveness of the fractions and combinations of cannabis compounds should be examined in GBM pre-clinical studies and clinical trials.

**Abstract:**

Glioblastoma multiforme (GBM) is the most lethal subtype of glioma. *Cannabis sativa* is used for the treatment of various medical conditions. Around 150 phytocannabinoids have been identified in *C. sativa*, among them Δ-9-tetrahydrocannabinol (THC) and cannabidiol (CBD) that trigger GBM cell death. However, the optimal combinations of cannabis molecules for anti-GBM activity are unknown. Chemical composition was determined using high-performance liquid chromatography (HPLC) and gas chromatography mass spectrometry (GC/MS). Cytotoxic activity was determined by XTT and lactate dehydrogenase (LDH) assays and apoptosis and cell cycle by fluorescence-activated cell sorting (FACS). F-actin structures were observed by confocal microscopy, gene expression by quantitative PCR, and cell migration and invasion by scratch and transwell assays, respectively. Fractions of a high-THC cannabis strain extract had significant cytotoxic activity against GBM cell lines and glioma stem cells derived from tumor specimens. A standard mix (SM) of the active fractions F4 and F5 induced apoptosis and expression of endoplasmic reticulum (ER)-stress associated-genes. F4 and F5 inhibited cell migration and invasion, altered cell cytoskeletons, and inhibited colony formation in 2 and 3-dimensional models. Combinations of cannabis compounds exert cytotoxic, anti-proliferative, and anti-migratory effects and should be examined for efficacy on GBM in pre-clinical studies and clinical trials.

## 1. Introduction

Glioma are primary brain tumors that arise from glial cells and account for ~80% of all malignant brain tumors [1]. According to the World Health Organization (WHO) classification, gliomas are classified from grade I (benign) to IV (malignant) [1]. They are also classified according to molecular parameters that define the tumor entity [2].

Among brain tumors, glioblastoma multiforme (GBM, WHO grade IV) is the most frequent, invasive, and lethal subtype [1,2]. Standard GBM therapies include maximal surgical resection followed by radio- and chemotherapy [3]. However, in recurrent or progressive GBM no standard of care is established, and treatments include surgery, re-irradiation, combined modality therapy, systemic therapies, and supportive care [3]. GBM often display a significant pathology and genetic heterogeneity within the tumor mass. In addition, there is evidence that GBM tumors contain cancer stem cells (GSCs). GSCs are characterized by self-renewal, multi-lineage differentiation potential, and the ability to generate xenografts that recapitulate the parental tumors [4,5]. GSCs have been implicated in tumor infiltration and resistance to radio- and chemotherapy as well as tumor recurrence. Altogether, these characteristics contribute to the high resistance of GBM cells to current standard therapies [6]. Consequently, effective therapies are urgently needed to improve the prognosis and quality of life for GBM patients.

*Cannabis sativa* is effective in the treatment of numerous medical conditions [7]. Around 600 constituents have been identified in *C. sativa*, among them hundreds of terpenes and more than 150 terpenophenolic compounds known as phytocannabinoids [8,9,10]. Recently, studies have shown that phytocannabinoids possess anticancer properties, including inhibition of cell proliferation, migration and angiogenesis, and induction of apoptosis in skin, prostate, lung, breast, and glioma cancer cells [11,12,13].

Phytocannabinoids were shown to trigger GBM cell death via different signal transduction pathways, including cell cycle arrest, oxidative stress, endoplasmic reticulum (ER)-stress, autophagy, and apoptosis [14,15]. One of the most abundant phytocannabinoids, Δ9-tetrahydrocannabinol (THC) [8], was shown to alter the balance between ceramides and dihydroceramides and subsequently activate ER-stress and apoptotic cell death [16]. The THC-induced ER-stress promoted autophagy, which was upstream of apoptosis, and the activation of this pathway was essential for the in vivo antitumor action of THC in tumor xenografts [17,18]. THC also induced G0-G1 phase arrest in GBM cells, but this effect of THC was not associated with apoptosis [19].

Interestingly, THC and cannabidiol (CBD) showed synergistic inhibition of cell proliferation in GBM cell lines [20]. Further, CBD was found to inhibit the invasiveness of GBM cells at sub-lethal concentrations [21]. In vivo, THC and/or CBD reduced tumor growth [14]. Cannabigerol (CBG) was also recently shown to reduce GBM cell viability and invasion in vitro [22].

Moreover, promising clinical evidence suggests effective cannabinoid-based treatments against GBM [15]. A pilot phase I clinical trial indicated that THC has a good safety profile. In this trial, administration of THC in two of nine GBM patients led to a decrease in tumor cell proliferation [23]. A THC:CBD mixture in combination with temozolomide (TMZ) was examined in preclinical trials [24] and in a placebo-controlled phase II clinical trial in GBM patients. It was announced that the THC:CBD plus TMZ group showed a higher rate of patient’s survival after 1 year compared to the control group treated by TMZ only [25]. These first results are promising and suggest translational research of cannabinoids for the treatment of GBM should be prioritized.

In many studies, it has been suggested that the natural combinations found in the plant are more effective than treatment with a single molecule [26,27,28]. Given the limited knowledge regarding cannabis activity against GBM, in this study, we identified cannabis extract fractions and combinations of cannabis molecules that are more active than single molecules. These were found to substantially reduce GBM cell viability and invasiveness in vitro and in 3D models.

## 2. Materials and Methods

### 2.1. Plant Extraction

A high-THC strain of *C. sativa* Dairy Queen (DQ) (IMC, Glil Yam, Israel) inflorescence was extracted as described previously [29]. Decarboxylation was done by heating the dry extract to 220 °C for 10 min. The decarboxylated extract was dissolved in methanol and filtered through a 0.45 µm syringe filter. Following evaporation, the weighted vials were resuspended in methanol to the desired concentrations.

### 2.2. Extract Fractionation

A flash chromatography apparatus equipped with a diode array detector was used to fractionize the decarboxylated crude extract. An Ecoflex C-18 80 g (Flash Pure, Buchi, Flawil, Switzerland; C-18, 80 µm spherical, max. pressure 180 psi) column was used for separation, with methanol and water as the mobile phase, as suggested by the manufacturer. The flow rate was set to 60 mL/min. The organic solvent (methanol) of each fraction was removed using a rotary vacuum evaporator at 30 °C. The remaining aqueous phase containing the compound of interest was further lyophilized to precipitate a dried powder. Each dried fraction tube was weighed separately and reconstituted using methanol to produce a solution with the required concentration and stored at −20 °C.

### 2.3. Chemical Analysis

High-performance liquid chromatography (HPLC 1260 Infinity II, Agilent) equipped with a Raptor ARC-18 LC-UV column (150 mm × 4.6 mm ID, pore size 2.7 µm) was used for chemical analysis as described in [29]. Isocratic separation was used with acetonitrile (25%) and water with 5mM ammonium formate, 0.1% formic acid (75%) at a constant flow rate of 1.5 mL/min. The sample concentration was 100 µg/mL, and injected volume was 5 µL. Cannabinoid profiles and fraction quantification were carried out in comparison to the standard calibration curves obtained by dissolving cannabinoid standards in methanol at a range of concentrations from 0–25 µg/mL. Gas chromatography with mass selective detector (GC/MS 8860 GC/5977B MSD, Agilent) equipped with 30 m, 0.25 mm ID, 5% cross-linked phenylmethyl siloxane capillary column (HP-5MS) with 0.25 μm film thickness was used for chemical analysis as described in [29]. Then, 10 μL of each fraction sample was transferred into a GC vial with 0.2 mL conical insert, dried under a gentle stream of nitrogen, and dissolved in 100 μL of hexane. The sample volume for injection was 1 μL. Helium was used as the carrier gas at a constant flow of 1.1 mL/s. An isothermal hold at 50 °C was maintained for 2 min, followed by a heating gradient of 6 °C/min to 300 °C, and the final temperature was held for 4 min. Peak assignments were performed using spectral libraries (NIST 14.0 and 17.0) and compared with MS data obtained from the injection of purchased standards (LGC Standards).

### 2.4. Standard/Material Preparation and Use

The cannabinoid standards (at a concentration of 1 mg/mL in methanol) used in this study included cannabidiol (CBD, Restek catalog no. 34011), cannabigerol (CBG, Restek catalog no. 34091), tetrahydrocannabivarin (THCV, Restek catalog no. 34100), cannabinol (CBN, Restek catalog no. 34010),a and delta-9-tetrahydrocannabidiol (Δ-9 THC, Restek catalog no. 34067). Inverse agonists (IA) to CB1 and CB2 used included AM251 (ab120088; Abcam) and SR144528 (ab146185; Abcam), respectively. The transient receptor potential ankyrin subtype 1 protein (TRPA1) blocker used was HC-030031 (ab120554; Abcam). Transient receptor potential vanilloid receptor 1 (TRPV1) and 2 (TRPV2) antagonists were SB-366791 (ab141772-5-B Abcam) and Tranilast (1098/10 Abcam), respectively. All IAs were dissolved in dimethyl sulfoxide (DMSO) at a concentration of 10 mM. Doxorubicin (D1515; Sigma Aldrich, St. Louis, MO, USA) served as a positive control in concentrations of 0.5 µg/mL on A172 cells and 50 µg/mL on U87. Temozolomid (TMZ, T2577; Sigma Aldrich, St. Louis, MO, USA) was tested as a positive control. Analytical grade methanol was used according to the indicated concentration of the treatment. Ultra-pure deionized water (MS grade) was used as received without further purification.

### 2.5. Cell Cultures

Human glioblastoma A172 (ATCC^®®^CRL-1620™) was cultured in DMEM (01-055-1A, Biological Industries, Israel) and U87MG (U87; ATCC^®®^HTB-14™) in EMEM (01-058-1A; Biological Industries, Israel). Both media were supplemented with 10% fetal bovine serum (FBS, 04-127-1A, Biological Industries, Beit Haemek, Israel), 1% Pen-Strep, 1% L-Glutamine, and 0.02% plasmocin (i.e., complete medium). Cells were incubated at 37 °C in a humidified atmosphere containing 5% CO2-95% air. Serum-free media (SFM) was composed of DMEM/F12 (01-170-1A; Biological Industries, Israel), including all the supplements above except for FBS. Neurosphere media (SpM) for A172 was composed of SFM with 2% B-27; for U87 the SpM used was serum-free EMEM without B27. For GSCs cultures all human materials were used in accordance with the policies of the Henry Ford Hospital Institutional Review Board. Generation of GSCs from fresh GBM specimens and their characterization have been recently described [30,31]. The GSCs were plated in neurosphere medium (DMEM-F12 1:1, glutamine 10 mM, HEPES buffer 10 mM, and sodium bicarbonate 0.025%) supplemented with EGF and bFGF (20 ng/mL).

### 2.6. Cell Viability Assays

Cells were seeded into 96-well plates at a density of 2 × 10^4^ per well (100 µL/well) in SFM (for A172) or complete medium (for U87) and were incubated at 37 °C overnight to allow attachment. The following day, cells were treated with plant extracts (*n* = 3), fractions, or cannabinoid standards in a volume of 100 µL/well at different concentrations. Solvents were used as a vehicle control, and doxorubicin was used as a positive control in all the biological assays. In experiments where CB1 or CB2 inverse agonists, TRPV1 or TRPV2 antagonists, and TRPA1 blocker were used, they were added along with the treatments at a concentration of 10 µM/mL in complete medium. Treated cells were incubated for 48 h at 37 °C. Subsequently, XTT reagents (2,3,-bis (2-methoxy- 4-nitro- 5-sulfophenyl)-5-[(phenylamino)- carbonyl]-2H- tetrazolium inner salt) (20-300-1000; Biological Industries, Israel) were added to the cells for 2 h at 37 °C in a humidified 5% CO_2_–95% air atmosphere. Absorbance was recorded by a Synergy H1 hybrid reader photometer (BioTek) at 490 nm with 650 nm of reference wavelength. Cell viability was estimated from the equation:

$$\%\;\mathrm{Cell}\;\mathrm{Viability}\;=\;100\;\times \;\frac{\left(A490-A650\right)\;\mathrm{of}\;\mathrm{treatment}}{\left(A490-A650\right)\;\mathrm{of}\;\mathrm{solvent}\;\mathrm{control}}$$

A490 and A650 are the absorbencies of the XTT colorimetric reaction. The absorbance of medium alone (blank) was subtracted from the readings. For dose-response assays, data points were connected by non-linear regression lines of the sigmoidal dose-response relation. GraphPad Prism version 6.1 (GraphPad Software Inc., San Diego, CA, USA) was employed to produce dose-response curves and determination of IC50 values.

For GSCs, cell viability was quantified by counting cells with trypan blue exclusion assay and using the lactate dehydrogenase cytotoxicity (LDH) assay kit. GSC neuro-spheres were disaggregated, and 10^5^/mL cells were plated in triplicates in 24 well plates. The cells were treated with the specific compounds, and cell death was determined at 24 and 48 h using the LDH assay kit according to the manufacturer’s instructions and as previously described [32]. The absorbance value of each sample was read at 490 nm, and cell death was determined compared to control untreated cells.

### 2.7. Colony Forming Assay

A172 or U87 cells were seeded into a 6-well plate at a density of 5 × 10^5^ in 3 mL of SFM or EMEM and were incubated at 37 °C overnight to allow attachment. The following day, cells were treated with the treatments in complete medium. After 24 h of incubation, cells were washed twice with 2 mL of PBS, harvested, and centrifuged for 3 min at 1400 rpm, then re-plated in serial cell-density dilutions in SpM for 48 h incubation. Colonies were imaged and counted by using an inverted microscope (Primo Vert Zeiss, Oberkochen, Germany). Groups of more than 20 cells were identified as a colony [33]. The percentage of colony in treatments was calculated out of the average number of colonies in the control at the highest cell concentration (6 × 10^4^).

### 2.8. Scratch-Wound Assay

A172 cells were seeded into a 96-well plate at a density of 2 × 10^4^ per well in 100 µL of complete medium. After 24 h, cells in each well were scratched perpendicularly across the center of the well with a 200 µL pipette tip to produce a cell-free area for investigating the ability of the cells to migrate and close the gap under different treatments. Immediately after scratching, 100 µL of treatment solution was added. Photos were taken at 0, 14, 20, and 36 h following scratching and the gap area was measured using ImageJ (version 1.53a). The scratch area, indicated by cells migrated into the scratch, was calculated as percent of the scratch area at time *x* from time 0:

$$\frac{\left(x\;h\;\mathrm{cell}\;\mathrm{free}\;\mathrm{area}\right)\times \;100\;}{\left(0\;h\;\mathrm{cell}\;\mathrm{free}\;\mathrm{area}\right)}$$

### 2.9. Transwell Assay

Cells were seeded into the upper chamber of a 24-well plate containing insert with an 8 µm pore size membrane (BD, Falcon Cat#353097), at a density of 5 × 10^4^ in 250 µL complete medium and were incubated at 37 °C for 60 min to recover. Cells were treated and incubated for 24 h followed by a viability test with resazurin (AR002, R&D Systems, Minneapolis, MN, USA). The inserts were taken out of the medium and the inner part was wiped using a cotton swab to remove the detached cells. The inserts were transferred into a new 24-well plate, fixated using 70% ethanol for 10 min and dried for 50 min, followed by staining in 400 µL of 0.2% crystal violet, washed with PBS, and dried for 5 min. Cells stained underneath the membrane were counted in an inverted microscope in 3–5 different fields.

### 2.10. Cell Staining

EasyProbes™ ActinRed 555 Stain was used for F-actin staining and Hoechst 33342 (ABP Biosciences, USA) for nuclei staining, according to the manufacturer’s instructions. Briefly, cells were seeded on confocal dishes (D35-20-1.5-N, Cellvis, Mountain View, CA, USA) at a density of 2 × 10^4^ in 500 μL of complete medium. Following 24 h incubation, treatments at different concentrations were added for 24 h. For the staining process, cells were washed with PBS and fixed with 3.7% formaldehyde solution in PBS and incubated at room temperature for 10 min followed by incubation in 0.1% Triton X-100 (T8787; Sigma Aldrich, St. Louis, MO, USA) for 5 min, washed again with PBS, and incubated in 1% BSA (A7284; Sigma Aldrich, St. Louis, MO, USA) solution for 30 min. Two drops of EasyProbes™ Actin and of Hoechst were applied to each of the samples for 20 min. Image acquisition was carried out using a Leica SP8 laser scanning microscope (Leica, Wetzlar, Germany), equipped with a diode laser with 405 nm and OPSL 552 nm laser, HC PL APO CS 10x/0.40 and HC PL APO CS2 63x/1.20 objectives (Leica, Wetzlar, Germany) and Leica Application Suite X software (LASX, Leica, Wetzlar, Germany). The number of F-actin was quantified as the number of filaments crossed once a line was drawn across the soma. The percentage of filaments in treatments was calculated out of the average number of filaments in the control.

### 2.11. Apoptosis Assay

Apoptosis for the A172 cell line was assessed using the aMEBCYTO Apoptosis Kit with Annexin V-FITC and PI (MBL, Enco, 4700). Staining was carried out according to manufacturer instructions. In brief, cells were seeded in a 6-well TC plate, at a density of 4 × 10^5^ cells in 2 mL of SFM per well. 24 h following seeding, cells were treated with treatments in complete medium for 48 h. After incubation, cells were harvested using trypsin and centrifuged for 5 min at 1600 rpm. Cell pellets were resuspended and washed twice with 1 mL of PBS. The cells in each sample were resuspended in 85 μL of Annexin binding buffer. Cells were stained using 10 μL of Annexin V-FITC solution and 5 μL of propidium iodide (PI) working solution followed by incubation in the dark at room temperature for 15 min. Then 400 μL of Annexin V binding buffer was added to each tube, and flow cytometry was performed using a Gallios flow cytometer (FACS). Cells were considered apoptotic if they were Annexin V+/PI- (early apoptosis) or Annexin V+/PI+ (late apoptosis). Live cells were defined as Annexin V-/PI-, and Annexin V-/PI+ as necrosis.

### 2.12. Cell Cycle Analysis

A172 cells were seeded in 6-well TC plate at a concentration of 4 × 10^5^ cells in 2 mL of SFM per well. After 24 h incubation, cells were treated with treatments in complete medium for 24 h. Methanol and doxorubicin were used as negative and positive controls, respectively. Cells were harvested and centrifuged for 5 min at 1600 rpm. Cell pellets were washed once with 1 mL of PBS and fixed with 70% cold ethanol overnight at −20 °C. The fixed cells were washed twice with 1 mL of PBS and stained with 250 μL of PI solution (50 μg/mL) containing RNase A (100 μg/mL) for 30 min in dark conditions. 250 μL of PBS was added to each tube, and the cells were analyzed using FACS.

### 2.13. Quantitative Real-Time PCR

Cells were seeded in a 6-well plate at a concentration of 1 × 10^6^ cells in 5 mL of SFM per well. After 24 h incubation, cells were treated with treatments in complete medium for 4, 12, and 24 h. Cells were harvested, and RNA was extracted using TRI reagent (T9424; Sigma Aldrich, St. Louis, MO, USA). RNA was reverse-transcribed in a total volume of 20 μL (PB30.11-10; PCR Biosystems IncqPCRBIO, Wayne, PA, USA) according to the manufacturer’s protocol. PCR was performed in triplicate using a StepOnePlus system (AB4346906, Applied Biosystems, Thermo Fisher Scientific, Waltham, MA, USA). The expression of each target gene was normalized to the expression of GAPDH mRNA using the 2^-ΔΔCt^ method, presenting the differences (∆) in threshold cycle (Ct) between the target gene and GAPDH gene. ΔCt=Ct target gene—Ct GAPDH. ΔΔCt=ΔCt treatment- ΔCt control. Experiments were repeated 3 times. The primers were: for CB2 (CNR2; Gene ID 1269) (forward) 5′- ATCATGTGGGTCCTCTCAGC -3′ and (reverse) 5′-GATTCCGGAAAAGAGGAAGG-3′; TRIB3 (Gene ID: 57761) (forward) 5′- GGTGCTTATCAGGTGCCAAG -3′ and (reverse) 5′- GTTGTCAGCTCAAGGATGCC -3′; ATF4 (Gene ID: 468) (forward) 5′- GGAAACCATGCCAGATGACC -3′ and (reverse) 5′- ACTTTCTGGGAGATGGCCAA -3′; CHOP (Gene ID: 1649) (forward) 5′- AGCAGAGGTCACAAGCACCT -3′ and (reverse) 5′- CTGGGGAATGACCACTCTGT -3′.

### 2.14. 3D Models

Hydrogels (AGFCH) included Sigma-Aldrich products and were prepared using alginate (W201502; Sigma-Aldrich, St. Louis, MO, USA) 22.5 mg/mL, gelatin (G9764; Bio-Basic, USA) 45 mg/mL, fibrinogen (F3879l; Sigma-Aldrich, St. Louis, MO, USA) 50 mg/mL, collagen (C9791; Sigma-Aldrich, St. Louis, MO, USA) 2.2 mg/mL and hyaluronic acid (O8185; Sigma-Aldrich, St. Louis, MO, USA) 2 mg/mL in PBS-glycerol solvent. The gel (AGFCH) was mixed with U87 cells at a concentration of 2 × 10^6^ cells per 400 μL gel. The solutions were mixed gently and transferred as 25 μL of gel solution to a 24-well plate. 3D models were cross-linked using CaCl_2_ (A610050; Bio-Basic, St. Louis, MO, USA) and thrombin (SRP6557; Sigma-Aldrich, St. Louis, MO, USA) for 5 min, after which the 3D structure was washed with PBS and then immersed in 1 mL of EMEM complete medium. Cells in the 3D structures were allowed to grow for 2 days, and then treatments were administered for 8 days (treatments were repeated every 2 days). Structures were stained using EasyProbe Hoechst, as described above. To assess cell viability, the 3D structure was digested using 200 μL of 0.05M sodium citrate (C3434; Sigma-Aldrich, St. Louis, MO, USA) and 0.05M EDTA (03-052-1A; Biological Industries, Beit Haemek, Israel) solution for 5 min. The solution was centrifuged for 4 min at 1700 rpm, the cell pellet was washed with PBS, and the Alamar Blue (resazurin, AR002; R&D Systems, Minneapolis, MN, USA) assay was performed. Groups of more than 20 cells were identified as a colony [33]. The percentage of colony in treatments was calculated out of the average number of colonies in the control.

### 2.15. Statistical Analysis

Results were presented as mean + SE of replicate analyses and were either representative of or included at least 2 independent experiments. Means of replicates were subjected to statistical analysis by a Tukey–Kramer test using the JMP statistical package (Ver. 14, SAS Inc, NC, USA) and considered significant when *p* ≤ 0.05.

## 3. Results

### 3.1. Determination of the Effect of C. sativa Extract and Fractions on A172 Cell Viability

**Figure 1:**
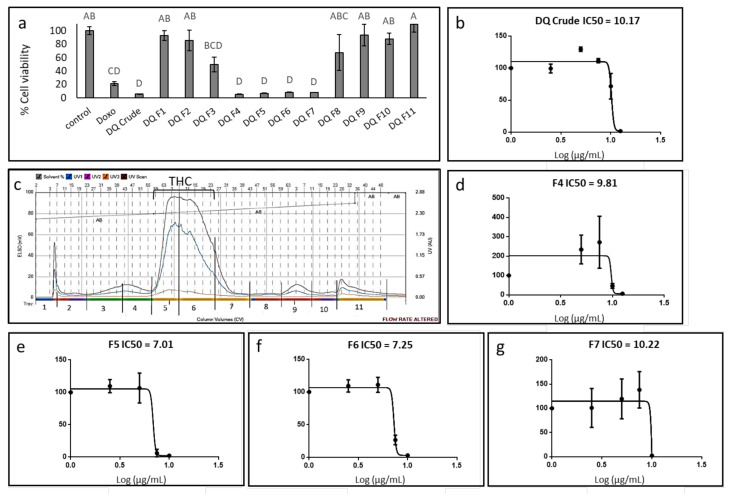
Cell viability of A172 cells following treatment with *C. sativa* Dairy Queen (DQ) crude extract and fractions. (**a**) Cell viability of A172 cells following treatment with crude extract and fractions F1-11 at a concentration of 12.5 μg/mL for 48 h. Cell viability was determined by XTT assay as a function of live cell number. Doxorubicin (Doxo 0.5 µg/mL) served as a positive control; control is the vehicle control (0.75% *v*/*v* methanol). Error bars indicate ± SE (*n* = 3). Levels with different letters are significantly different from all combinations of pairs according to Tukey–Kramer honest significant difference (HSD; *p* ≤0.05). (**b**) Cell viability of A172 cells following treatment with *C. sativa* DQ crude extract at different concentrations. The IC50 values were calculated from 5P logistic curve fit using GraphPad Prism version 6.1. (**c**) Flash chromatography profile of *C. sativa* DQ crude extract. Fractions were collected and designated as F1–F11. The approximate range of the THC peak is shown. (**d**–**g**) Cell viability of A172 cells following treatment with *C. sativa* DQ fractions F4, 5, 6, 7 at different concentrations. The IC50 values were calculated from 5P logistic curve fit using GraphPad Prism version 6.1. Error bars indicate ± SE (*n* = 3).

The methanol extract of high-THC *C. sativa* strain DQ was found to be cytotoxic to the GBM A172 cell line (Figure 1a), with a calculated IC50 of 10.17 µg/mL following treatment for 48 h (Figure 1b). Phytocannabinoid content in the crude extract was 55.2% (Table 1). To identify the active compounds of the DQ extract, fractionation was performed using flash chromatography (Figure 1c), and the cytotoxic activity of fractions was examined on the A172 cell line. Four of the 11 fractions (F4–F7) showed significant cytotoxic activity at the examined concentration, resulting in ~90% cell death (Figure 1a). Treatments with F3 or F8 showed moderate cytotoxic activity (50% and 30% cell death, respectively; Figure 1a). F1, F2, F9, and F10 exhibited only minor cytotoxic activity, resulting in approximately ~10% cell death, whereas treatment with F11 did not lead to cell death. Rather, it caused minor (~110%) cell proliferation (Figure 1a).

The calculated IC50 of F4, fractionated prior to the emergence of the THC peak from flash chromatography (Figure 1c), was 9.81 µg/mL (Figure 1d). Among the fractions allocated at the THC peak, designated F5–F7 (Figure 1c), F5 was the most active with IC50 of 7.01 µg/mL (Figure 1e). IC50 values of F6 and F7 were 7.25 and 10.22 µg/mL, respectively (Figure 1f,g). Notably, at sub-lethal concentrations, the crude extract and some of the fractions, especially F4 and F7, led to cell proliferation (~200% and ~150%, respectively; Figure 1b,d,g respectively). TMZ did not lead to substantial A172 cell death even at relatively high concentrations (i.e., up to 50 µg/mL; Supplementary Figure S1).

F4 and F5 were also active on the U87 cell line, however, at higher concentrations in comparison to their activity on the A172 cell line (Supplementary Figure S2a). Doxorubicin at relatively high concentrations (i.e., up to 50 µg/mL) was only moderately active on this cell line under the examined conditions (Supplementary Figure S2b).

### 3.2. Determination of the Chemical Composition of the Active Fractions

The composition of active fractions, i.e., F4–F7, was chemically characterized using HPLC (Table 1). F5 and F6 were similar in content and included mainly THC (Table 1), F7 included mainly THC and cannabichromene (CBC), and F4 contained mainly cannabigerol (CBG) (Table 1).

Further analysis was carried out on the most active THC fraction, F5, and the most active non-THC fraction, F4. The F4 and F5 terpenes content (terpenes constituted ~32% of crude extract) determined by GC/MS is presented in Supplementary Table S1.

### 3.3. Determination of Activity of the Standard Mixes of F4 and F5

**Figure 2:**
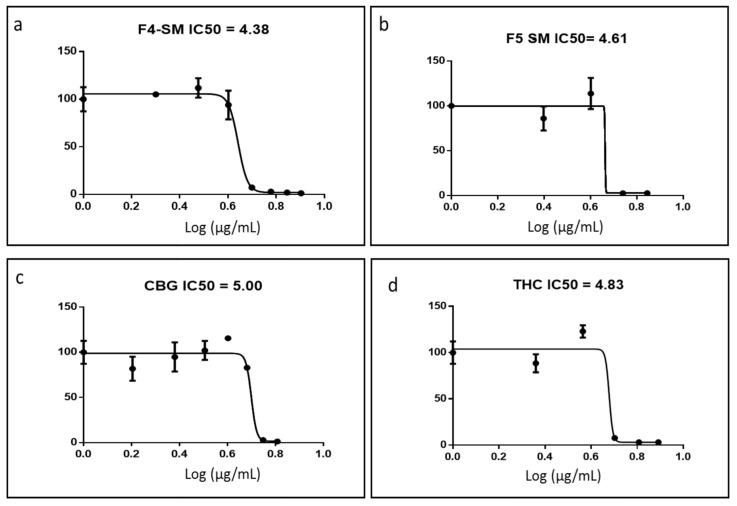
Cell viability of A172 cells following treatment with F4-SM (**a**), F5-SM (**b**), CBG (**c**), and THC (**d**) at different concentrations for 48 h. Cell viability was determined by XTT assay as a function of live cell number. The IC50 values were calculated from 5P logistic curve fit by GraphPad Prism version 6.1. Error bars indicate ± SE (*n* = 3).

In order to confirm the active compositions of F4 and F5, IC50 values of the phytocannabinoid standard mix (SM) of each fraction were examined and calculated. SM is the mix of phytocannabinoid standards equivalent of the primary phytocannabinoids in Table 1, at the appropriate percentages to be as close to F4 and F5 as possible. F4-SM had an IC50 of 4.38 µg/mL (Figure 2a), which was lower than that of F4 (9.81 µg/mL). F5-SM showed an IC50 value of 4.61 µg/mL (Figure 2b), again lower than F5 (7.01 µg/mL). F4-SM showed a significant reduction of cell proliferation at sub-lethal concentrations (~110% of cell proliferation in comparison to ~200% in F4 treatments; Figure 2a). However, F5-SM led to similar levels of cell proliferation at sub-lethal concentrations in comparison to F5 (~115% and ~110%, respectively; Figure 2b). Interestingly, CBG, the primary molecule of F4, was less active than F4-SM (IC50 of 4.38 and 5.00 µg/mL for F4-SM and CBG, respectively; Figure 2c). Similarly, THC, the primary molecule of F5, was less active than F5-SM (IC50 of 4.61 and 4.83 µg/mL for F5-SM and THC, respectively; Figure 2d).

F4-SM and F5-SM were also active on the U87 cell line, however, at higher concentrations in comparison to active concentrations on A172 cells (Supplementary Figure S2a). F4-SM activity was higher than F4 on U87 cells (Supplementary Figure S2a).

### 3.4. Determination of the Effect of F4-SM or F5-SM Treatments on Apoptosis

**Figure 3:**
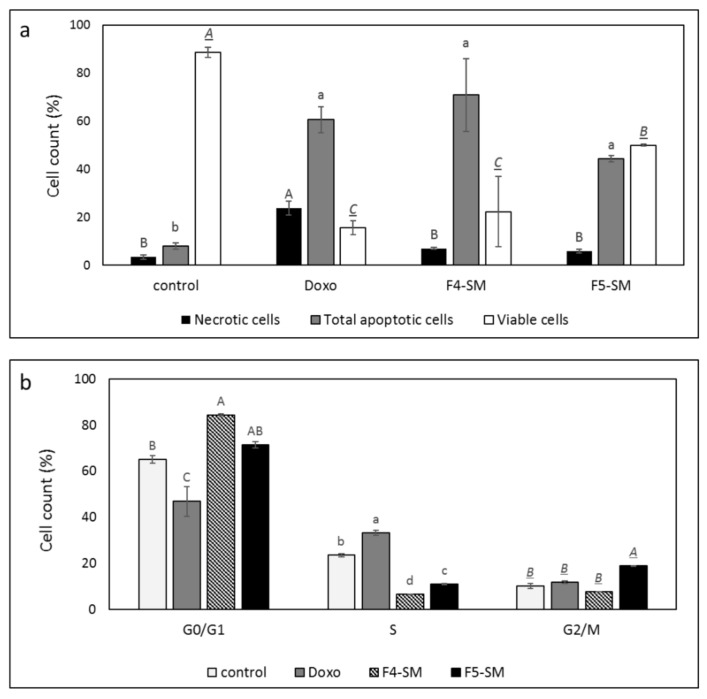
(**a**) Percentage of viability, apoptosis, or necrosis in A172 cells following treatment with F4 SM (10 µg/mL) or F5 SM (10 µg/mL) for 48 h. (**b**) Percentage of A172 cells in G0/G1, G2/M, and S phase following treatment with F4-SM (10 µg/mL) or F5-SM (10 µg/mL) for 24 h. 10^4^ cells were analyzed per treatment. Control is vehicle control (1% *v*/*v* methanol) and doxorubicin (Doxo, 0.5 µg/mL) served as positive control. The treated cells were harvested and analyzed in FACS following annexin V-FITC and PI staining. Error bars indicate ± SE (*n* = 3). Levels with different letters are significantly different from all combinations of pairs according to the Tukey–Kramer honest significant difference (HSD; *p* ≤ 0.05).

Since the SM of the fractions were more cytotoxic than the extract fractions, we also determined the effect of treatments with F4-SM or F5-SM on cell apoptosis. Treatment with F4-SM or F5-SM for 48 h led to 70.8% and 44.3% cell apoptosis, respectively, in comparison to 8.0% apoptosis in the vehicle control and 60.6% in doxorubicin positive control (Figure 3a; Supplementary Figure S3). Moreover, treatments of A172 with F4-SM and F5-SM led to a lower percentage of necrotic cells (6.9% and 5.8%, respectively) compared to doxorubicin (23.8%) but higher than in the methanol control (3.4%; Figure 3a; Supplementary Figure S3).

### 3.5. Determination of the Effect of F4-SM or F5-SM Treatments on Cell Cycle Arrest

F4-SM treatment of A172 for 24 h led to an increase in the percentage of cells in the G1 phase of the cell cycle (84.5%) in comparison to the control (vehicle) treatment (65.2%; Figure 3b; Supplementary Figure S3). F5-SM treatment led to significant enrichment in the percentage of cells in the G2-M phase (18.8%) in comparison to 10.2% in the control, 11.6% in the doxorubicin, and 7.6% in F4-SM (Figure 3b; Supplementary Figure S3). Both F4-SM and F5-SM treatments led to a reduction in the percentage of S phase cells (6.5% and 11.0%, respectively) in comparison to the vehicle control (23.5%; Figure 3b; Supplementary Figure S3).

### 3.6. Determination of the Involvement of CB1 and CB2 Receptor Inverse Agonists, TRPA1 Receptor Blocker and TRPV1 and TRPV2 Receptors Antagonists on Cytotoxic Activity

**Figure 4:**
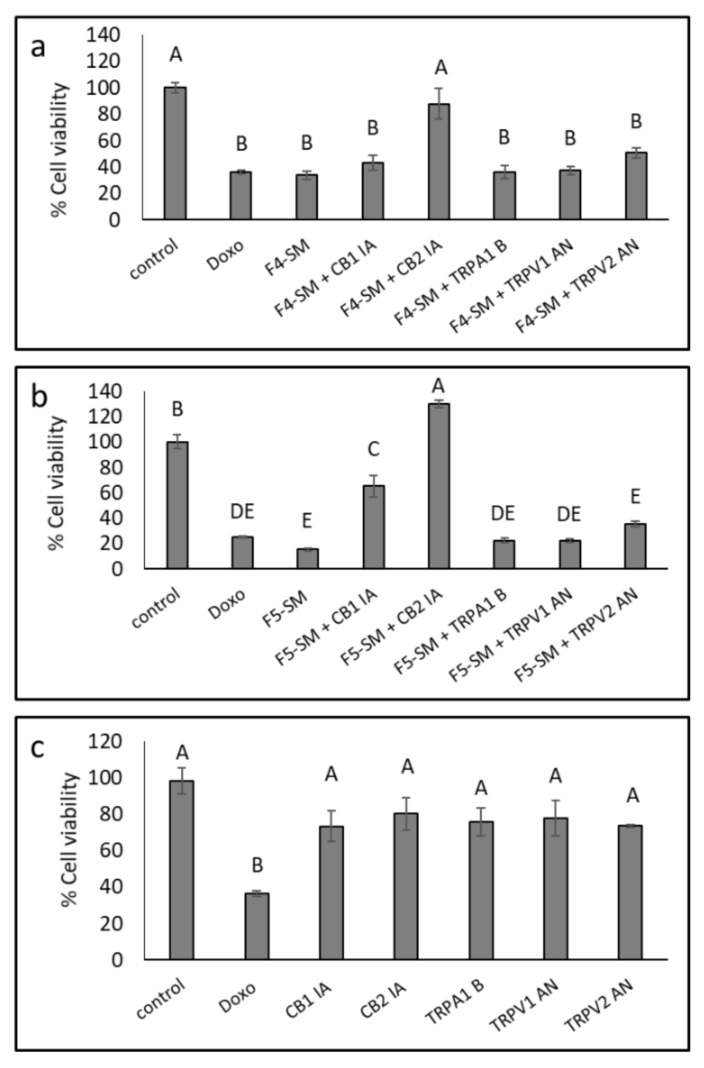
Cell viability of A172 cells following treatment with F4-SM (**a**) and F5-SM (**b**), with or without CB1 and CB2 inverse agonists (IA), a TRPA1 blocker (B), and TRPV1 or TRPV2 antagonists (AN) for 48 h. (**c**) The effect of IA, B, or NA on cell viability. Cells were treated with F4-SM (12 µg/mL) or F5-SM (10 µg/mL) in addition to the receptors IA, B, or AN (10 µM). Cell viability was determined by XTT assay as a function of live cell number. Doxorubicin (Doxo, 0.5 µg/mL) served as a positive control. Control is vehicle control (1.1% *v*/*v* methanol + 1% DMSO). Error bars indicate ± SE (*n* = 3). Levels with different letters are significantly different from all combinations of pairs according to the Tukey–Kramer honest significant difference (HSD; *p* ≤ 0.05).

Since the SM of the fractions were more cytotoxic than the extract fractions, we also determined the effect of adding CB1 or CB2 IA, TRPV1, or TRPV2 antagonists (AN) or TRPA1 blocker (B) on F4-SM and F5-SM activity. A172 cells were treated with F4-SM and F5-SM with or without the IA, AN, or B. In the presence of CB2, the cytotoxic effect of F4-SM was significantly reduced (87.9% vs. 33.8% viable cells with or without CB2 IA, respectively; Figure 4a). In the case of F5-SM, the addition of CB2 IA led to cell proliferation (129.9% vs. 15.3% viable cells with or without CB2 IA, respectively; Figure 4b). The addition of CB1 IA to F5-SM treatment led to inhibition of the cytotoxic activity, but to a lesser extent than CB2 IA (65.1% vs. 15.3% cell viability for F5-SM with or without CB1 IA, respectively; Figure 4b).

Treatments with F4-SM in the presence of TRPV1 or TRPV2 AN, TRPA1 B or CB1 IA, or F5-SM treatment with TRPV1, TRPV2 AN, and TRPA1 B did not significantly affect the cytotoxicity of the treatments (Figure 4a,b). CB1 and CB2 IA, TRPV1 and TRPV2 AN, and TRPA1 B reduced A172 cell viability to a minor, non-significant extent (Figure 4c).

CB2 (*CNR2*) was expressed in A172 cells, and its expression was reduced with the F5-SM treatments and induced with F4-SM treatment (Table 2). However, we could not detect CB1 expression in these cells.

### 3.7. Determination of Gene Expression of ER-Stress Related Genes

**Figure 5:**
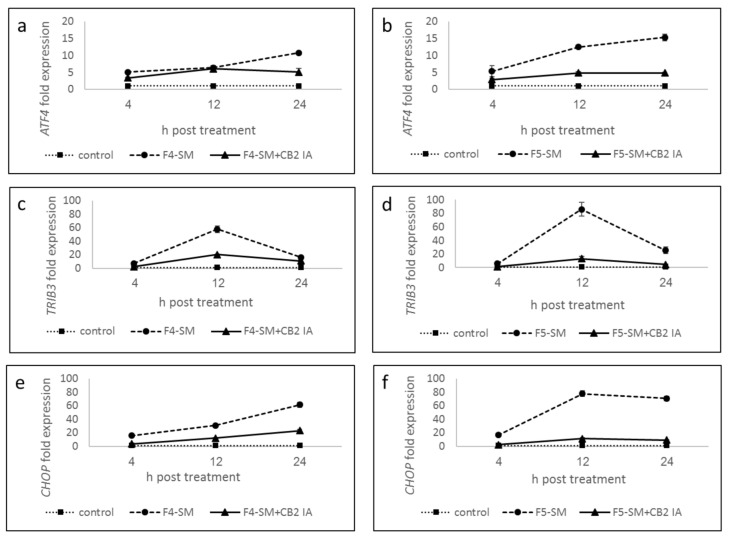
Quantitative PCR determination of the RNA steady-state level in A172 cell line of *ATF4* (**a**,**b**), *TRIB3* (**c**,**d**), and *CHOP* (*DDIT3-3*) (**e**,**f**) genes, after treatment with F4-SM or F5-SM (10 µg/mL) relative to control. Control is vehicle control (1.2% *v*/*v* methanol). Gene transcript values were determined by quantitative PCR. Mean values ± SE are shown (*n* = 3). Statistical analysis of gene expression results are in Supplementary Table S2.

To explore the possible induction of ER-stress by F4-SM and F5-SM treatments, we determined the expression of *ATF4*, *TRIB3,* and *CHOP* (*DDIT3-3*) genes. F4-SM and F5-SM treatments substantially induced these gene expressions (Figure 5; Supplementary Table S2), with the highest *ATF4* expression at 24 h (Figure 5a,b; Supplementary Table S2). *TRIB3* expression was the highest at 12 h in both treatments (Figure 5c,d; Supplementary Table S2). *CHOP* expression was the highest at 24 h with F4-SM or 12 and 24 h with F5-SM treatments (Figure 5e,f; Supplementary Table S2). Co-treatment with CB2 IA considerably reduced induction by F4-SM or F5-SM of all gene expression (Figure 5; Supplementary Table S2).

### 3.8. Determination of the Effect of SM Treatments on GSC Viability

**Figure 6:**
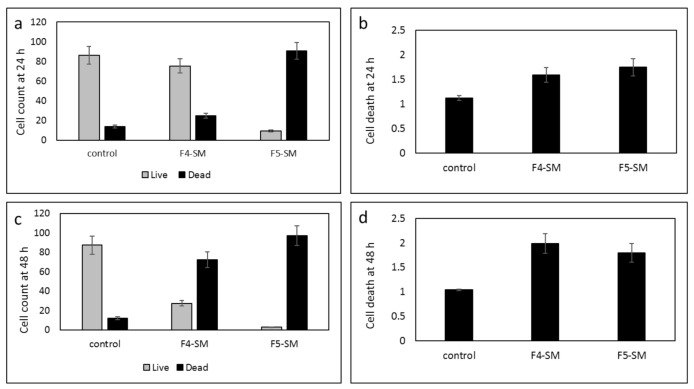
Cell viability of GSCs following treatment with F4-SM or F5-SM (10 µg/mL) for 24 h (**a**,**b**), F4-SM or F5-SM for 48 h (**c**,**d**). Live/dead cell count was determined by trypan blue exclusion assay (**a**,**c**); LDH assay was used to determine cell death relative to vehicle control (**b**,**d**). Error bars indicate ± SE (*n* = 3).

GSCs exhibit resistance to chemotherapy and radiation therapy and are implicated in tumor infiltration and recurrence. Therefore, identifying treatment that targets these cells is of great importance. To analyze the cytotoxic effects of F4-SM and F5-SM on GSC, we employed neurosphere cultures (GSC-1) that were generated from a GBM primary tumor. The GSCs were maintained as spheroids, and their self-renewal, differentiation, and tumorigenic abilities were validated as previously reported [30,31]. We found that treatment of GSC-1 with F4-SM and F5-SM at a concentration of 10 µg/mL induced strong cytotoxic effects that were already observed after 24 h (Figure 6a,b). Treatment of GSCs with F4-SM for 48 h further increased cell death (Figure 6c,d). However, F5 exerted a stronger cytotoxic effect already after 24 h, and after 48 h, the majority of the treated cells exhibit cell death (Figure 6).

### 3.9. Determination of the Effect of Fraction or SM Treatments on Cell Motility

**Figure 7:**
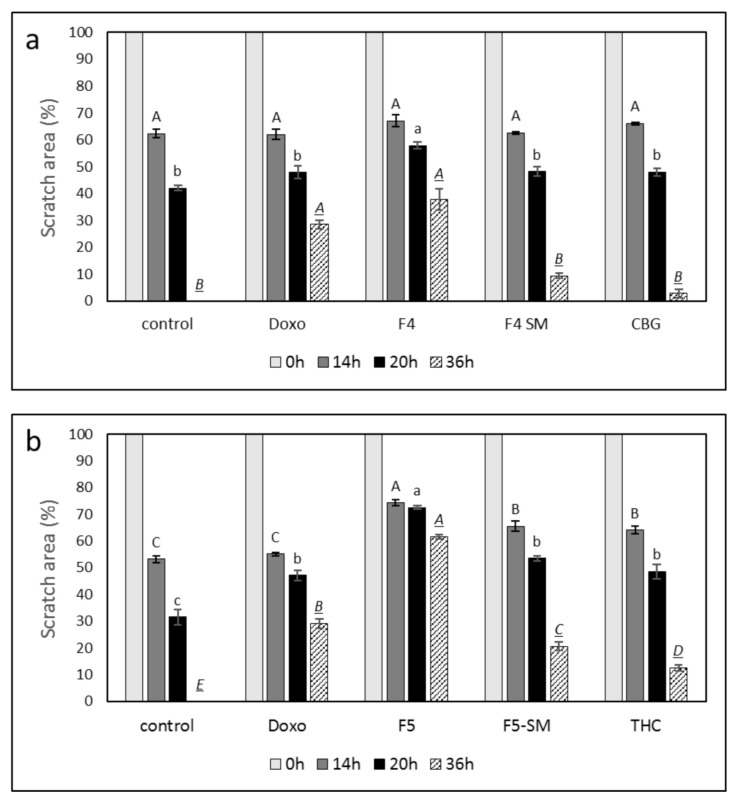
Effect of F4, F5, F4-SM, or F5-SM on A172 cell migration. Cells were treated with (**a**) F4 (20 µg/mL); F4-SM (11.5 µg/mL) or CBG (9.2 µg/mL) (**b**) F5 (20 µg/mL); F5-SM (11.5 µg/mL) or THC (10.5 µg/mL). Control is vehicle control (1.15% *v*/*v* methanol). Doxorubicin (Doxo, 0.5 µg/mL) served as a positive control. Percent scratch area is presented as mean; error bars indicate ± SE (*n* = 3). Levels with different letters are significantly different from all combinations of pairs according to the Tukey–Kramer honest significant difference (HSD; *p* ≤ 0.05).

To examine the ability of the fractions and SM to attenuate cancer cell motility, the effects of F4, F5, and their corresponding SM on cell migration at sub-lethal concentrations were examined using scratch assays. Treatments of the A172 cell line with F4 or F5 for 36 h led to significant inhibition of cell migration (37.7% and 61.6%, respectively) in comparison to the vehicle control (Figure 7; Supplementary Figure S4). Treatment with F4-SM or F5-SM at concentrations corresponding to those in the extract fractions led to less inhibition of cell migration, 9.5% and 20.5%, respectively, in comparison to the control (Figure 7; Supplementary Figure S4). Treatment with the primary molecules of F4 or F5, i.e., CBG or THC, led to even less inhibition of cell migration (2.8 and 12.5%, respectively, in comparison to the control). Doxorubicin treatment also inhibited cell migration (28.8% in comparison to control), but this was lower than F4 or F5 (Figure 7; Supplementary Figure S4).

### 3.10. Determination of the Effect of Fractions Treatments on the Cytoskeleton in A172 Cells

**Figure 8:**
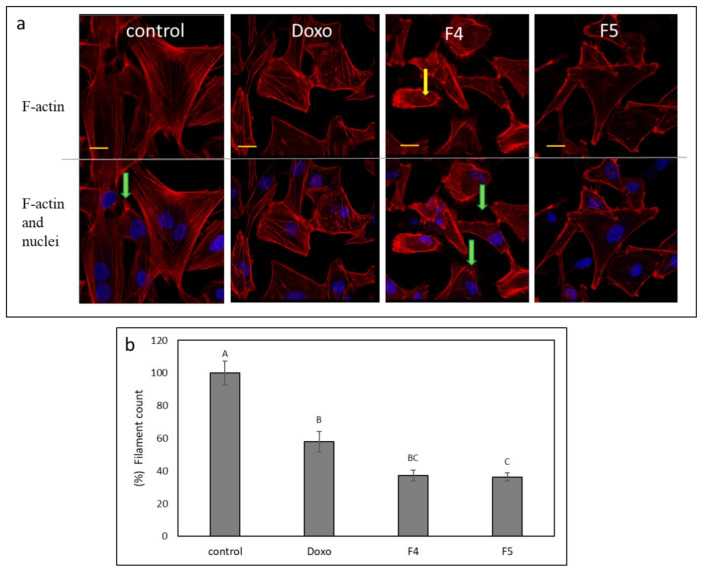
(**a**) Representative examples of confocal images of A172 cells following treatment with F4 (12 µg/mL) and F5 (10 µg/mL) for 24 h. F-actin (EasyProbes™ ActinRed 555 Stain, red stain) and nuclei (Hoechst, blue stain) were stained. Control is vehicle control (1.15% *v*/*v* methanol). Doxorubicin (Doxo, 0.5 µg/mL) served as a positive control. Scale bars = 20 µm; *n* = 3. Yellow arrows point to the disintegration of F-actin filaments visualized as characteristic spots; green arrows show F-actin filopodia protruding from cells. (**b**) The number of F-actin filaments that crossed a line drawn across the soma in each of the treatments. The percent number of filaments is presented as mean; error bars indicate ± SE (*n* = 5). Levels with different letters are significantly different from all combinations of pairs according to the Tukey–Kramer honest significant difference (HSD; *p* ≤ 0.05).

F4 and F5 treatments were the most effective treatments for the inhibition of cell migration (Figure 7). Hence, we examined the changes in cytoskeleton structures in A172 for these treatments. F-actin filaments of cells treated with vehicle control formed organized and distinct networks with few protruding filopodia (Figure 8a). However, the F-actin network disappeared in cells treated with F4 and was replaced with diffused dot-like actin structures (Figure 8a yellow arrows, Fig 8b). Furthermore, F4 treatment led to the induction of F-actin filopodia compared to the control (Figure 8a, green arrows). The F-actin network also disappeared in cells treated with F5 (Figure 8a,b). However, F5 treatment reduced the appearance of filopodia in cells in comparison to F4 (Figure 8a). Doxorubicin effect on F-actin structures was less pronounced in comparison to that of F4 or F5 (Figure 8a,b).

### 3.11. Determination of the Effect of F4 and F5 Treatments on Cell Invasion

**Figure 9:**
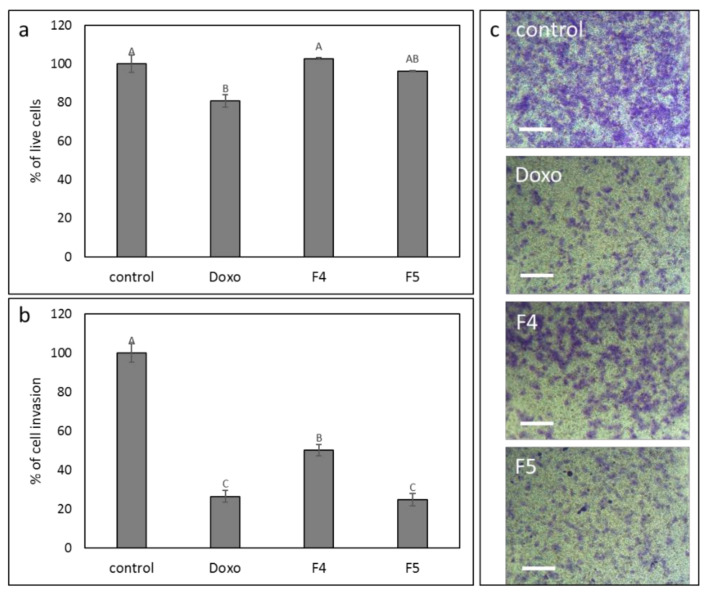
The effect of the F4 and F5 treatments on A172 cell invasion. The vertical movement of A172 cells across the 8 µm pore size membrane, following treatment with F4 (12 μg/mL) and F5 (10 µg/mL) for 24 h. Error bars indicate ± SE (*n* = 3). Levels with different letters are significantly different from all combinations of pairs according to the Tukey–Kramer honest significant difference (HSD; *p* ≤ 0.05). (**a**) Percentage of cell viability; (**b**) percentage of invading cell in comparison to control; (**c**) examples for images of cells that invaded the membrane at 24 h, following crystal violet staining. Scale bars = 500 µm. Doxorubicin (Doxo, 0.5 µg/mL) served as a positive control. Control is vehicle control (0.6% *v*/*v* methanol).

Since F4 and F5 treatments were the most effective treatments for inhibition of cell migration (Figure 7), we have examined the effects of these treatments on cell invasion. At mostly sub-lethal concentrations (Figure 9a), F4 and F5 treatments substantially reduced cell invasion in a transwell assay, F5 to a greater extent (43.6 vs. 18.6%) relative to vehicle control (Figure 9b). As a positive control, doxorubicin treatment reduced cell invasion by 22.5% in comparison to the vehicle control (Figure 9b), in agreement with [34]. Figure 9c shows examples of cells that invaded the membrane at 24 h for each treatment.

### 3.12. Determination of the Effect of Fractions or SM Treatments on Colony Formation

**Figure 10:**
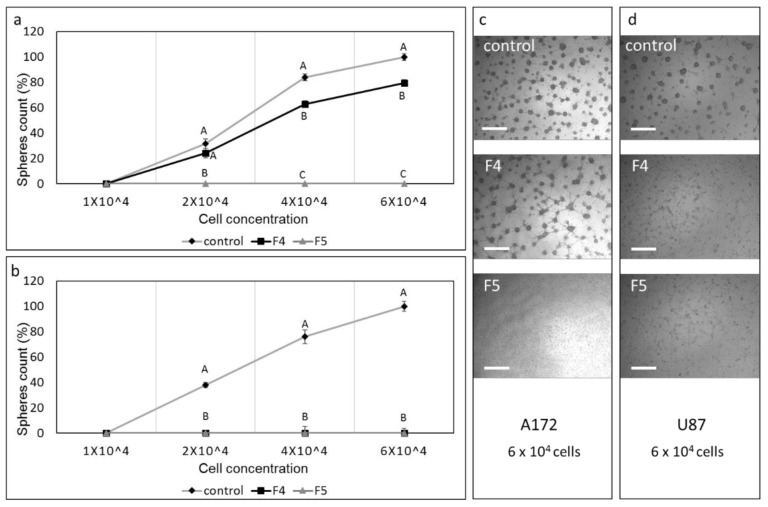
Assay for colony formation of A172 and U87 cells following treatments with F4 and F5. Percentage of colonies (out of the average number of colonies in the control at the highest cell concentration [6 × 10^4^]) following treatment of (**a**) A172 with F4 (20 µg/mL) or F5 (16.5 µg/mL) and (**b**) U87 cells with F4 (12 µg/mL) or F5 (10 µg/mL) for 24 h. Cells were sorted to live cells, and these were seeded under conditions that promote neurosphere formation. Error bars indicate ± SE (*n* = 3). Levels with different letters are significantly different from all combinations of pairs according to the Tukey–Kramer honest significant difference (HSD; *p* ≤ 0.05). (**c**–**d**) Examples of images used to count colonies in 6 × 10^4^ cell cultures. Scale bars = 500 µm. Control is vehicle control (0.5% *v*/*v* methanol).

To examine the effect of F4, F5 on colony formation, cells were sorted following treatments to live cells, and these live cells were re-seeded and allowed to form colonies. Treatment with F4 in A172 cells reduced colony formation to a small but significant extent (Figure 10a,c). However, treatment with F5 completely abolished colony formation in A172 (Figure 10a,c). In the U87 cell line, F4 and F5 treatments led to complete abolishment of colony formation at all examined cell concentrations (Figure 10b,d).

### 3.13. Determination of the Effect of Fractions or SM Treatments on Colony Formation in 3D Structures

**Figure 11:**
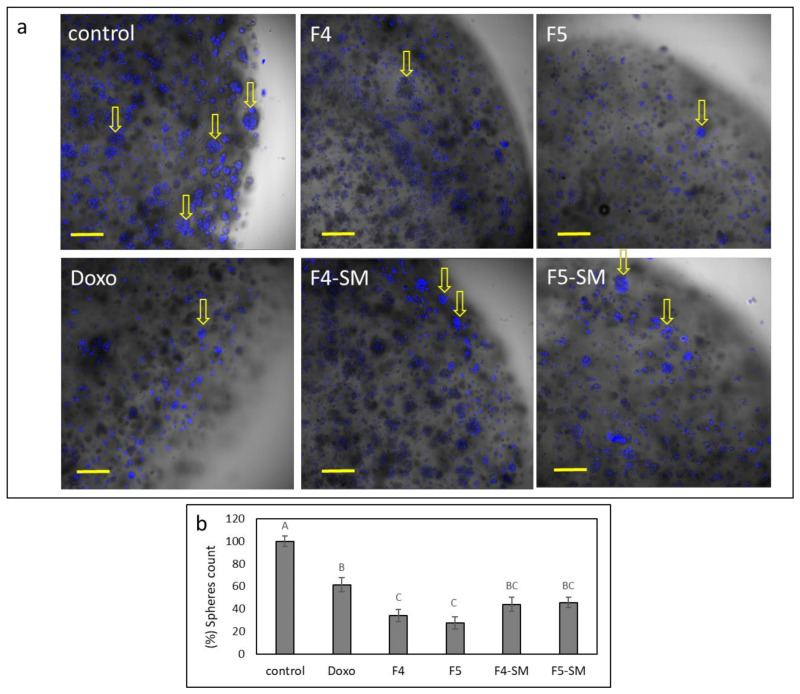
(**a**) Examples for representative confocal images of U87 cells following treatment with F4 (20 µg/mL), F5 (16.5 µg/mL), F4-SM (12.5 µg/mL), and F5-SM (10 µg/mL), for 8 d. Doxorubicin (Doxo, 2.0 µg/mL) served as a positive control. Control is vehicle control (2.0% *v*/*v* methanol). Overlay of brightfield and Hoechst staining for nuclei (blue color) is shown in five concurrent optical sections; scale bars = 200 µm; *n* = 3. Arrows point to some of the colonies that formed. (**b**) Percentage of the number of colonies in each of the treatments (out of the average number of colonies in the control), presented as mean; error bars indicate ± SE (*n* = 3). Levels with different letters are significantly different from all combinations of pairs according to the Tukey–Kramer honest significant difference (HSD; *p* ≤ 0.05).

To examine the effect of the fractions on colony formation in 3D structures, U87 cells were seeded in extra cellular matrixes, forming 3D droplets, and were treated with F4, F5, F4-SM, F5-SM, or doxorubicin. Multiple colonies were formed in the control (~20 colonies; Figure 11a,b). However, in the F4 and especially in F5 treated structures, cells were dispersed, and fewer colonies were formed (Figure 11a,b). The number of live cells in these structures was also substantially reduced (Supplementary Table S3). Treatments with F4-SM or F5-SM at concentrations corresponding to those in the extract fractions reduced colony formation, similarly to doxorubicin, but to a somewhat lesser extent than the fraction treatments (Figure 11a,b).

## 4. Discussion

Combinations of cannabis compounds identified in extract fractions of a high-THC cannabis strain have significant cytotoxic activity against GBM cell lines A172 and U87. The activity of F4 and F5 was higher than that of the crude extract, suggesting that fractionation in this case, as in other cases [28,29,35], may increase extract activity. This adds to a growing list of studies that show that fractionation and determination of active fractions (in this case, F4 and F5) allow the identification of molecules and/or their combinations from cannabis that are active for particular action [35]. In this study, the two fractions were composed of different combinations of phytomolecules, with CBG and THC as the most abundant in F4 and F5, respectively. Several studies have demonstrated CBG anticancer activity, including in mouse melanomas, human oral epithelioid carcinoma (KB) cells, human breast carcinoma, and colorectal cancer cells [36]. Moreover, THC and CBG were shown to induce apoptosis in GBM cells [14,15,17,22]. The SM of the fractions described here, F4 or F5, were more active than their primary molecule alone, i.e., CBG or THC, respectively. This further solidifies the notion that the natural combination of compounds found in the plant may be more effective than a single molecule [26,27,28]. However, the F4-SM or F5-SM were more cytotoxic to GBM cells than the corresponding extract fraction.

As noted above, a clinical pilot study of THC treatment in GBM patients showed that THC can be administered safely and has anti-proliferative effects [23]. However, we found that at sub-lethal concentrations, some of the THC-predominant treatments (e.g., the crude extract) led to cell proliferation in the A172 cell line. In agreement with our results, it was previously demonstrated that treatment of glioblastoma cell line U373-MG and lung carcinoma cell line NCIH292 with nanomolar concentrations of THC promoted cell proliferation. This proliferation was dependent on the activity of metalloprotease and epidermal growth factor receptor (EGFR) and tumor necrosis factor α-Converting Enzyme (TACE/ADAM17) that mediated EGFR transactivation [37]. The basis for the proliferative effect of the THC-predominant treatments in our study might be similar. Taken together, it can be concluded that the biological responses of GBM cancer cells to THC are critically dependent on drug concentrations [37]. Importantly, this proliferative effect at low concentrations should be taken into consideration, as it raises concerns for clinical use: It might be difficult to control treatment with high doses only, continually, to ensure cell death rather than cell proliferation in patients.

Treatment with F4-SM or F5-SM led to cell apoptosis in A172 cells. In many cases, apoptosis resulted from the activation of cell cycle arrest [38]. CBG and THC were indeed suggested to alter GBM cell cycle, but in the case of THC, this effect on the cell cycle was not associated with cell apoptosis [19,22]. However, the F4-SM and F5-SM treatments led to only minor differences in the cell cycle in comparison to the control. Another cellular condition that leads to apoptosis is ER-stress; THC induction of ER-stress in cancer cells, GBM included, is well established [14,15,17]. *ATF4* is a transcription factor transiently induced after treatment with ER stressors [39]. In turn, ATF4 induces genes that act in amino acid synthesis and transport, protein synthesis and secretion, and antioxidant stress responses [40]. ATF4 also induces *CHOP-10* (*GADD153/DDIT-3*) expression, which is a transcription factor with roles in the induction of cell death [39]. Another protein associated with ER stress is TRIB3. TRIB3 was found to facilitate ER-stress-dependent apoptosis via the NF-κB pathway [41].

In our study, F4-SM and F5-SM treatments induced *ATF4*, *CHOP-10* (*GADD153/DDIT-3*), and *TRIB3* gene transcription, suggesting that indeed the F4-SM and F5-SM treatments induce cell death via ER stress. In agreement, THC induced an ER-stress response in human and mouse glioma cells that promoted autophagy and apoptosis via TRIB3, inducing expression of *ATF4*, *CHOP*, and *TRIB3* genes [14,17,42]. By binding to CHOP, TRIB3 represses CHOP/ATF4 transactivation leading to downregulation of its own transcriptional induction [43]. In both F4-SM and F5-SM treatments, *TRIB3* expression peaked at 12 h and reduced at 24 h. This expression profile might be a result of the negative feedback loop impaired by TRIB3 [41,43]. CBG was also shown to stimulate apoptosis and to up-regulate *CHOP* mRNA in colorectal cancer (CRC) cells [44].

GBM has a dismal prognosis that is partly attributed to the presence of GSCs that exhibit self-renewal abilities and resistance to radiation and chemotherapy. Indeed, one of the barriers to the successful treatment of GBM is the eradication of the GSC subpopulation. Although GSCs represent only a small percentage of the tumor cells in GBM, they are implicated in tumor recurrence. The potent antitumor effect of the F4-SM and F5-SM compounds on GSCs demonstrated in this study, in addition to the pronounced effects of these compounds on GBM cell lines, emphasizes the potential of these compounds as novel and potent therapeutic agents for the treatment of GBM.

The antitumor effects of phytocannabinoids are mediated at least partially by targeting the endocannabinoid system [14,15]. In the present study, CB2 IA significantly blocked F4-SM and F5-SM cytotoxic activity. Hence, the CB2 receptor might be involved to some extent in F4-SM and F5-SM activity. Indeed, CB2 expression in A172 cells was evident and was reduced with F5-SM and induced with F4-SM treatments. Previously, CB2 receptor expression was found to correlate with tumor malignancy grades in GBM cell lines and to be highly expressed in malignant tissue biopsies compared to normal tissue [14] and in spheroids of patient-derived cell lines [22]. Both THC and CBG were also found previously to be partial agonists of CB2 receptors [45]. Importantly, CB2 IA substantially blocked in our study induction by F4-SM and F5-SM of *CHOP*, *ATF4*, and *TRIB3* gene expression, further supporting the idea that the CB2 receptor plays a role in these SMs activity on GBM cells.

CB1 IA blocked the activity of F5-SM, however, we could not detect CB1 gene expression in the A172 cells. Since the CB1 IA used here (AM251) sometimes also acts as an inverse agonist of the CB2 receptor (e.g., EC50 value of 650 ± 30 nM; [46]), it might be that the reduction in F5-SM activity in the presence of AM251 was a result of its IA activity on the CB2 receptor. CB1 has been shown to be expressed in GBM cell lines and spheroids [e.g., 22]. However, there is inconsistent data regarding CB1 receptor expression in GBM in general [14].

THC also activates TRPA receptors [45], and CBG is also a TRPV1 and TRPA1 agonist [45]. Nevertheless, in our study, neither the TRPA1 blocker nor TRPV1 or TRPV2 antagonists reduced F4-SM or F5-SM activity, suggesting TRPA1, TRPV1, and TRPV2 are not involved in the cytotoxic activity of these compounds on GBM cells.

In contrast to the improved cytotoxic activity of the SMs in comparison to the extract fractions, the SMs and primary phytomolecules (i.e., CBG or THC) were less effective than the extract fractions in inhibiting cell migration in scratch assays. Again it might be that the other molecules present in F4 and F5, in addition to phytocannabinoids (e.g., terpenes), facilitate the inhibition of cell migration. F-actin integrity is important for cell motility [47]. Treatment with either F4 or F5 disrupted F-actin organization and caused the disappearance of actin filaments, although treatment with F4 also led to increased filopodia. Disturbances in F-actin organization may indeed lead to a reduction in GBM cell motility [48]. However, filopodia are used by cancer cells to invade surrounding tissue [49]. Despite this, F4 treatment inhibited rather than enhanced cell invasion in vitro, possibly by suppressing other components necessary for invasion. The invasiveness of GBM is partially due to the elevated migratory potential of individual cells into surrounding and distant brain tissue. The migrating cells contribute to recurrent glioblastomas and therapeutic resistance [5,50]. It was previously suggested that tumor invasion might be a plausible target for GBM intervention, and specific anti-migratory compounds should be considered in addition to conventional radio- and chemotherapy [51].

Another important observation in this study was the substantial reduction of colony formation by F4 and F5. GBM colonies developed in vitro were denoted as spheres [52] and suggested to have characteristics of stem cells, including the ability to self-renew and differentiate to daughter cells of different phenotypes [52]. Importantly, treatment of 3D structures of U87 cells with F4 and F5 substantially reduced colony formation, with the extract fractions more effective than SMs. The higher effectiveness of the fractions may be due to the fraction’s ability to reduce cell viability and motility considerably; both associated with colony and neurosphere formation [53]. Interestingly, U87 cells were less sensitive to the cytotoxic effect of F4 or F5 but more sensitive to the F4 effect on sphere formation in comparison to A172 cells. U87 and A172 are GBM cell lines that harbor different genetic mutations [54,55]. For example, A172 cells carry a p53 mutation, whereas U87 cells express a wild-type p53. A172 cells are incapable of invading de-epithelialized tracheas, whereas U87 cells do so via increased activity of matrix metalloprotease-3 (MMP-3). In addition, A172 and U87 differ to some extent in their tumorigenic activity in immunosuppressed mice and their response to progesterone (P4) [54,55]. It is possible that the differences between U87 and A172 cells identified here are a result of their different genetic background. However, this hypothesis has to be examined in future research while unveiling F4 and F5 mechanisms of activity on GBM. Nevertheless, as neurosphere formation is associated with the high resistance of GBM cells to current standard therapies [6], F4 and F5 treatments may have the potential to decrease GBM cell invasion and therapy resistance. However, this suggestion will be further examined in vivo.

## 5. Conclusions

F4-SM and F5-SM were found to have high cytotoxic activity against GBM cell lines and GSCs in vitro and to induce expression of genes associated with ER-stress. F4 and F5 were also demonstrated to inhibit cell migration, invasion, and colony/neurosphere formation in GBM cells in vitro. The different treatments also affected the cell cytoskeleton. It was previously suggested that a combined anti-proliferative and anti-migratory treatment improved the response of GBM in vivo [56]. The cannabis compounds identified here convey this combination of effects and should be further examined regarding the treatment of GBM in pre-clinical studies and clinical trials.

## Figures and Tables

**Table 1:** Phytocannabinoid percentage of total phytocannabinoids in the crude DQ strain extract, and the F4–F7 fractionated from the extract. CBC, cannabichromene; CBD, cannabidiol; CBDV, cannabidivarin; CBDVA, cannabidivarinic acid; CBG, cannabigerol; CBGA, cannabigerolic acid; CBN, cannabinol; THC, Δ9–tetrahydrocannabinol; THCA, Δ9-tetrahydrocannabinolic acid; THCV, Δ9–tetrahydrocannabivarin.

Fraction/Compound	CBC	CBD	CBDV	CBDVA	CBG	CBGA	CBN	THC	THCA	THCV
Crude	5.0	0.1	<0.1	<0.1	4.0	0.1	1.8	87.4	0.5	1.0
F4	-	5.8	-	-	80.3	-	2.8	-	-	11
F5	-	-	-	-	3.7	-	4.6	91.7	-	-
F6	-	-	-	-	2.2	-	0.9	96.9	-	-
F7	23.5	-	1.8	0.9	1.3	-	1.9	68.9	1.7	-

**Table 2:** Quantitative PCR determination of the RNA steady-state level of CB2 receptor (*CNR2*) gene in A172 cell line, after treatment with F4-SM or F5-SM (10 µg/mL) for 12 h relative to control. Methanol (control) treatment served as a solvent (vehicle) control. Gene transcript values were determined by quantitative PCR. Mean values ± SE are shown (*n* = 3).

Treatment	CB2 Receptor (*CNR2*) Relative Expression
F4-SM	2.32 ± 0.49
F5-SM	0.47 ± 0.09

## Data Availability

Data supporting reported results can be found in Supplementary data.

## Supplementary Materials

The following are available online at https://www.mdpi.com/article/10.3390/cancers13071720/s1, Figure S1: Cell viability of A172 cells following treatment with temozolomide (TMZ) at different concentrations for 48 h. Figure S2: Cell viability of U87 cells following treatment with (a) F4, F5, F4-SM, or F5-SM and (b) doxorubicin (Doxo) at indicated concentrations for 48 h. Figure S3: (A) Annexin V-FITC and PI staining for determining the proportion of viable (Q4), apoptotic (Q2 and Q3 for late and early apoptosis, respectively), or necrotic cells (Q1). (B) Example of FACS output following PI staining to determine the stages of cell cycle arrest. Figure S4: Examples of pictures taken for estimating the effectiveness of treatment F4 (20 µg/mL), F4-SM (11.5 µg/mL), F5 (20 µg/mL), F5-SM (11.5 µg/mL), or doxorubicin (Doxo, 0.5 µg/mL) on recovered area of confluent monolayers of the A172 cell line at 0, 14, 20, and 36 h. Methanol (control) treatment served as a solvent (vehicle) control. Table S1: Percentage of terpenes out of total terpenes in F4 and F5 fractionated from DQ extract. Table S2: Statistical analysis for quantitative PCR determination of the RNA steady state level in the A172 cell line of *ATF4*, *TRIB3*, and *CHOP* (*DDIT3-3*) genes, after treatment with F4-SM or F5-SM (10 µg/mL) relative to control, presented in Figure 6. Table S3: Cell viability of U87 cells from 3D structures following treatment with F4, F5, F4-SM, and F5-SM at the indicated concentrations for 48 h.
