# Supplementary material for: Specific Compositions of Cannabis sativa Compounds Have Cytotoxic Activity and Inhibit Motility and Colony Formation of Human Glioblastoma Cells In Vitro
Source: Cancers (Basel). 2021 Apr 5;13(7):1720. doi: 10.3390/cancers13071720 (PMC8038598; doi:10.3390/cancers13071720)
Supplement: Supplementary file 1 [file cancers-13-01720-s001.pdf]

cancers

Supplementary Materials: Specific Compositions of *Cannabis sativa* Compounds Have Cytotoxic Activity and Inhibit Motility and Colony Formation of Human Glioblastoma Cells In Vitro

Hadar Peeri, Nurit Shalev, Ajjampura C. Vinayaka, Rephael Nizar, Gila Kazimirsky, Dvora Namdar, Seegehalli M. Anil, Eduard Belausov, Chaya Brodie, Hinanit Koltai

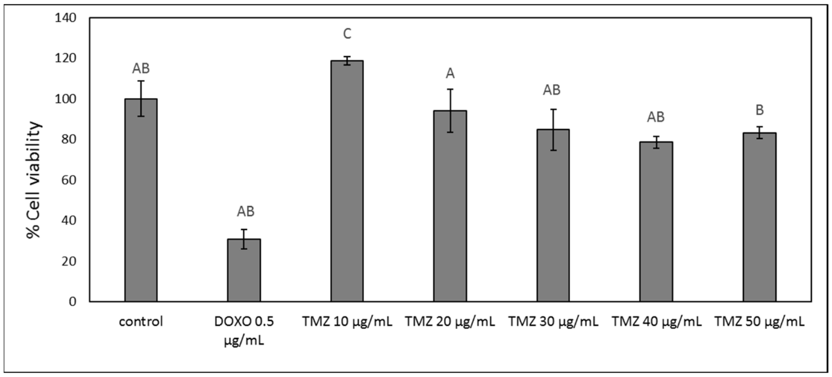

Figure S1. Cell viability of A172 cells following treatment with temozolomide (TMZ) at different concentrations for 48 h. Cell viability was determined by XTT assay as a function of live cell number. Doxorubicin (Doxo 0.5 µg/mL) served as positive controls; control is solvent (vehicle) control (0.5% *v*/*v* DMSO). Error bars indicate ± SE (n = 3). Levels with different letters are significantly different from all combinations of pairs by Tukey–Kramer honest significant difference (HSD; $p \leq 0.05$).

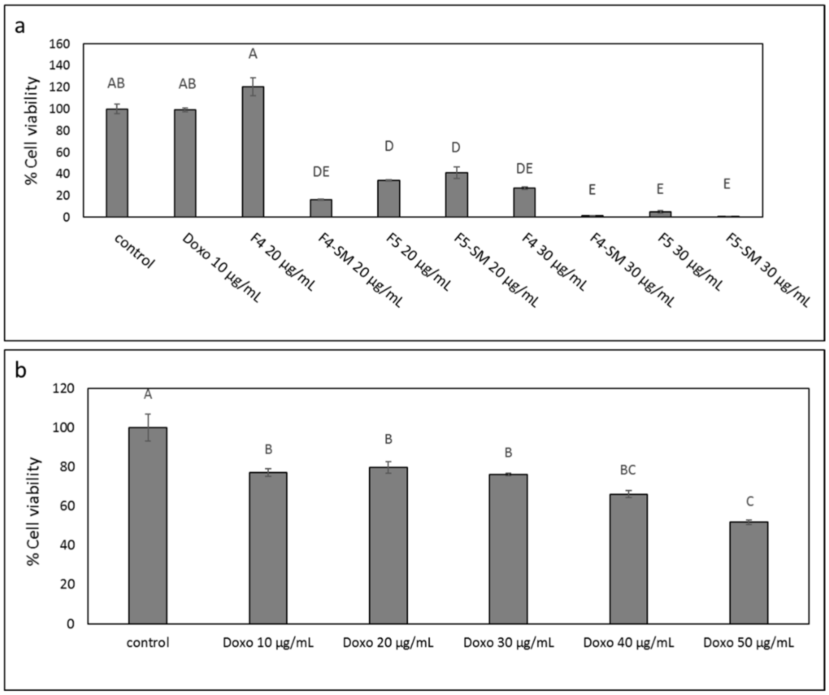

Figure S2. Cell viability of U87 cells following treatment with (**a**) F4, F5, F4-SM, or F5-SM and (**b**) doxorubicin (Doxo) at indicated concentrations for 48 h. Cell viability was determined by XTT

assay as a function of live cell number. Control is solvent (vehicle) control (1.5% *v*/*v* methanol). Error bars indicate ± SE (n = 3). Levels with different letters are significantly different from all combinations of pairs according to the Tukey–Kramer honest significant difference (HSD; $p \leq 0.05$).

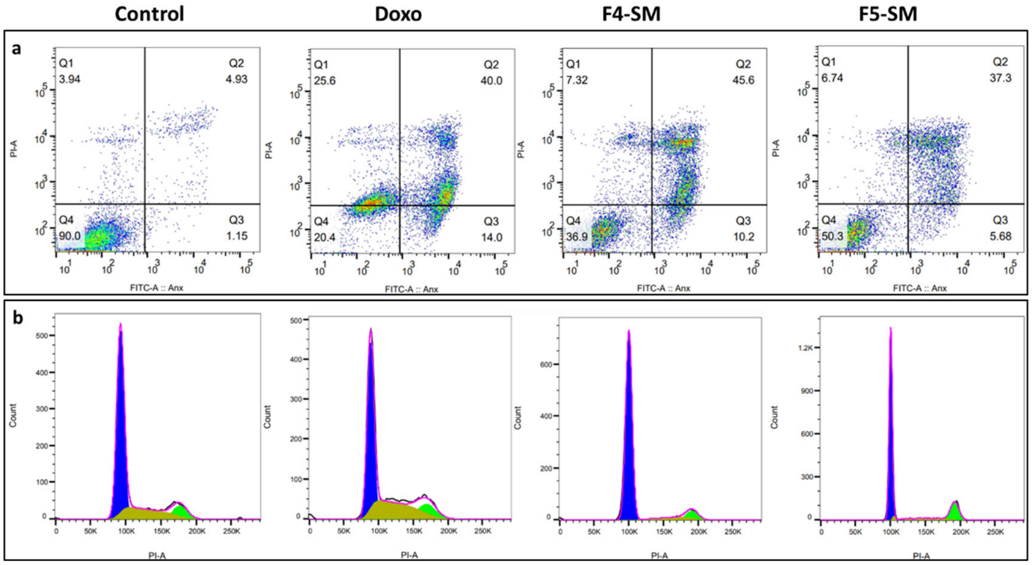

Figure S3. (**a**) Annexin V-FITC and PI staining for determining the proportion of viable (Q4), apoptotic (Q2 and Q3 for late and early apoptosis, respectively), or necrotic cells (Q1). (**b**) Example of FACS output following PI staining to determine the stages of cell cycle arrest. Treatments included F4-SM (10 µg/mL) or F5-SM (10 µg/mL) on the A172 cell line for 24 h for cell cycle and 48 h for apoptosis. Doxorubicin (Doxo, 0.5 µg/mL) served as a positive control. Methanol (control) treatment served as a solvent (vehicle) control.

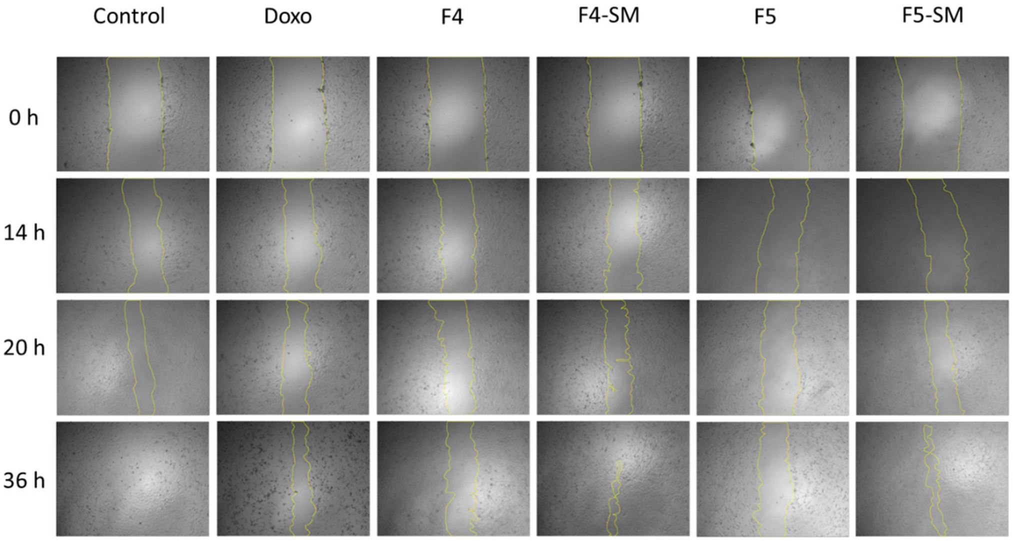

Figure S4. Examples of pictures taken for estimating the effectiveness of treatment F4 (20 µg/mL), F4-SM (11.5 µg/mL), F5 (20 µg/mL), F5-SM (11.5 µg/mL), or doxorubicin (Doxo, 0.5 µg/mL) on recovered area of confluent monolayers of the A172 cell line at 0, 14, 20, and 36 h. Methanol (control) treatment served as a solvent (vehicle) control.

Table 1. Percentage of terpenes out of total terpenes in F4 and F5 fractions of the DQ extract.

**Terpenes (%)**	**F4**	**F5**
α -Bisabolol	1.38	0.65
α-Eudesmol	62.35	19.29
γ-Eudesmol	18.13	23.78
9-Isopropyl-1-methyl-2-methylene-5-oxatricyclo [5.4.0.0(3,8)]undecane		0.74
Agarospirol	1.80	
Azulemanthanol	5.43	
Bulnesol		15.46
Caryophyllene oxide		1.57
α -Cedrene		0.53
β-Selinene		2.32
Guaiol	10.92	26.90
Nerolidol		6.33
Selina-3,7(11)-diene		2.43

Table S2. Statistical analysis for quantitative PCR determination of the RNA steady state level in A172 cell line of *ATF4*, *TRIB3*, and *CHOP* (*DDIT3-3*) genes, after treatment with F4-SM or F5-SM (10 μg/mL) relative to control, presented in Figure 5. Control is vehicle control (1.2% *v*/*v* methanol). Letters with similar style (uppercase, lowercase, italic, and/or bold letters) were compared for each gene and time post treatment.

	**Treatment/Time Post Treatment**	**4 h**	**12 h**	**24 h**
ATF4	Control (for F4-SM)	**C**	*B*	C
	F4-SM	**A**	*A*	A
	F4-SM+CB2 IA	**B**	*A*	B
	Control (for F5-SM)	**b**	*c*	c
	F5-SM	**a**	*a*	a
	F5-SM+CB2 IA	**ab**	*b*	b
TRIB3	Control (for F4-SM)	**B**	*C*	C
	F4-SM	**A**	*A*	A
	F4-SM+CB2 IA	**B**	*B*	B
	Control (for F5-SM)	**a**	*b*	b
	F5-SM	**a**	*a*	a
	F5-SM+CB2 IA	**a**	*b*	b
CHOP	Control (for F4-SM)	**C**	*C*	C
	F4-SM	**A**	*A*	A
	F4-SM+CB2 IA	**B**	*B*	B
	Control (for F4-SM)	**b**	*b*	c
	F5-SM	**a**	*a*	a
	F5-SM+CB2 IA	**b**	*b*	b

Table S3. Cell viability of U87 cells from 3D structures following treatment with Figure 4. F5, F4-SM, and F5-SM at the indicated concentrations for 48 h. Cell viability was determined by 3D structure disintegration and cell dispersal following an Alamar Blue (resazurin) assay. Control is vehicle control (3% *v*/*v* methanol). Means (n = 3) with different letters are significantly different from all combinations of pairs according to the Tukey–Kramer honest significant difference (HSD; $p \leq 0.05$).

**Treatment**	**Mean (% of Live Cells)**
Control	98.52±0.08 [a]
Doxorubicin 2 μg/mL	37.22±3.08 [c]
F4 20 μg/mL	11.98±0.44 [d]
F4-SM 12.5 μg/mL	17.39±1.14 [d]
F5 16.5 μg/mL	12.32±0.02 [d]
F5-SM 10 μg/mL	72.81±0.05 [b]
